# Twenty-Five–Year Follow-Up of the MDDC1 Family: A *LMNA* Gene Variant Associated With Dilated Cardiomyopathy With Variable Skeletal Muscle Involvement

**DOI:** 10.1161/CIRCGEN.125.005528

**Published:** 2026-04-01

**Authors:** Teresa Maria Capovilla, Manuela Iseppi, Salvatore Distaso, Marta Gigli, Matteo Dal Ferro, Michele Moretti, Annamaria Iorio, Antonella Mancinelli, Marco Merlo, Luisa Mestroni, Alessia Paldino, Gianfranco Sinagra

**Affiliations:** 1 Center for Diagnosis and Treatment of Cardiomyopathies, Cardiovascular Department, Azienda Sanitaria Universitaria Giuliano-Isontina (ASUGI), member of the European Reference Network for Rare, Low-Prevalence, or Complex Diseases of the Heart (ERN GUARD-Heart), University of Trieste, Italy. (T.M.C., M.G., M.D.F., M. Merlo., A.P., G.S.).; 2 Division of Cardiology, Ospedale Santa Chiara, Azienda Sanitaria Universitaria Integrata del Trentino (ASUIT), Trento, Italy (M.I., M. Moretti).; 3 Cardiology Unit, Health District of Mesagne, Mesagne, Italy (S.D.).; 4 Division of Cardiology, Cardiovascular Institute and Adult Medical Genetics Program, University of Colorado Anschutz Medical Campus, Aurora (L.M.).; 5 Cardiology Unit, Cardiovascular Department, Azienda Socio Sanitaria Territoriale (ASST) Papa Giovanni XXIII, Bergamo, Italy (A.I., A.M.).

**Keywords:** cardiomyopathy, dilated, genetics, muscular dystrophy, Emery-Dreifuss, muscular dystrophies, limb-girdle

Variants in *LMNA*, which encodes the nuclear envelope lamin A/C proteins, are well-established causes of genetic dilated cardiomyopathy (DCM), identified in up to 8% of affected individuals.^1^ These variants are often associated with electrical conduction defects and variable skeletal muscle involvement, which typically precede cardiac symptoms by several years. Brodsky et al^2^ described one of the first families with *LMNA*-related DCM and skeletal myopathy (MDDC1 [name of the family described in the original report]), characterized by a heterozygous single-nucleotide deletion of the *LMNA* gene (*c.960del* formerly annotated as *c.959delT*), resulting in a frameshift and a premature stop codon (p.Arg321Glufs*159).

Here, we report the 25-year follow-up of the MDDC1 family, originally described in 2000, providing new insights into the longitudinal progression of the cardiac and skeletal muscle phenotype associated with this *LMNA* variant. All living affected family members were reevaluated in 2025 through clinical and instrumental examinations. Genetic testing was repeated using Sanger sequencing of exon 6 of *LMNA*, confirming the presence of the variant in all affected and its absence in unaffected members.

Among the 14 family members, 5 carried the *c.960del* variant, all of whom developed clinically relevant manifestations (Figure [A] through [C]). None of the third-generation individuals carrying the *LMNA c.960del* variant had children, and no new carriers were identified during the 25-year follow-up. At first observation, the proband (II.5) was diagnosed with a pure DCM phenotype at age 30 years and died at 32 years due to sudden cardiac death. Patient II.1 presented an overlapping arrhythmic DCM phenotype and a mild limb-girdle muscular dystrophy (LGMD); he underwent heart transplantation at 37 years for end-stage DCM and has remained clinically stable with mild myopathic symptoms.

**Figure.**
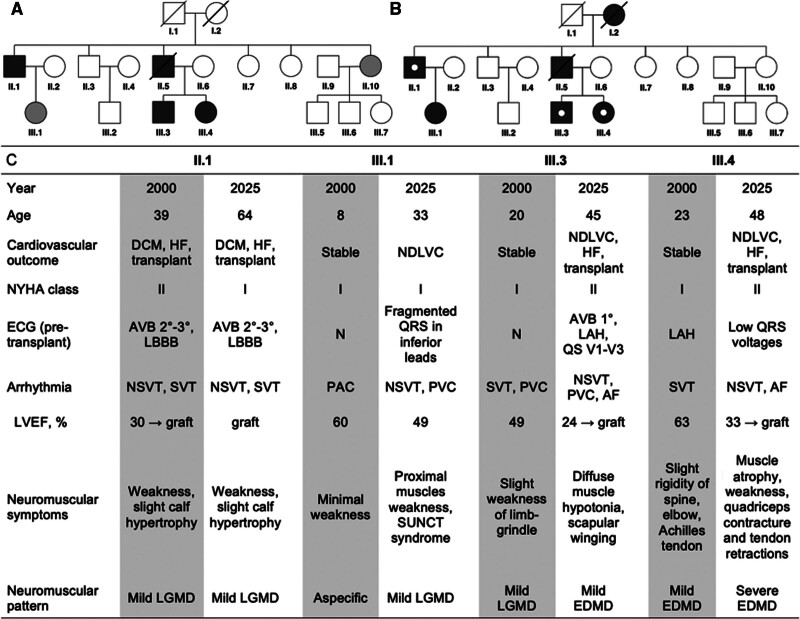
Pedigree and clinical summary of the MDDC1 (name of the family described in the original report) family carrying the *LMNA c.960del* (p.Arg321Glufs*159) variant. A and **B** show the family pedigree in 2000 (**A**) and after 25 years of follow-up in 2025 (**B**). Individuals are labeled according to generation and pedigree number. Filled squares (men) and circles (women) indicate individuals carrying the LMNA c.960del variant, all of whom developed clinically manifest disease. Open symbols represent unaffected individuals without the variant. Shaded symbols indicate unknown or limited information on disease and genotype status. A white dot inside a symbol denotes a history of heart transplantation. **C**, Main clinical and instrumental findings of affected family members at the time of the original report and at the most recent evaluation, including age, cardiovascular outcome, New York Heart Association (NYHA) functional class, electrocardiographic findings, arrhythmic profile, left ventricular ejection fraction (LVEF), and neuromuscular manifestations. AF indicates atrial fibrillation; AVB, atrioventricular block; DCM, dilated cardiomyopathy; EDMD, Emery-Dreifuss muscular dystrophy; HF, heart failure; LAH, left anterior hemiblock; LBBB, left bundle branch block; LGMD, limb-girdle muscular dystrophy; NDLVC, non-dilated left ventricular cardiomyopathy; NSVT, nonsustained ventricular tachycardia; PAC, premature atrial contractions; PVC, premature ventricular contractions; SUNCT, short-lasting unilateral neuralgiform headache attacks with conjunctival injection and tearing; and SVT, supraventricular tachycardia.

Patients III.3 and III.4 initially showed mild myopathic phenotypes in the absence of overt cardiac involvement, with a non-dilated left ventricle (LV) and preserved left ventricular ejection fraction (LVEF). Patient III.4 differed from the others by presenting mild atrophy of the biceps brachii, peroneal, and supraspinatus muscles, consistent with Emery-Dreifuss muscular dystrophy (EDMD). Patient III.1, examined at 4 years old, showed no clinical symptoms.

During follow-up, heterogeneous progression of cardiac and skeletal muscle disease was observed (Figure [C]). Patient II.1 has exhibited a stable post-transplant course to date, without major complications and with mild myopathic symptoms.

Patient III.3 developed a sudden and rapidly progressive form of non-dilated left ventricular cardiomyopathy with isolated global LV systolic dysfunction. At age 21 years, he was first hospitalized for heart failure (HF) during an episode of atrial fibrillation, with evidence of severe LV dysfunction (LVEF 24%). Within 6 months, the patient developed refractory HF and underwent successful heart transplantation. He remains clinically stable with preserved graft function but developed EDMD with diffuse muscle hypotonia, scapular winging, and mild proximal weakness.

Patient III.4 slowly developed a non-dilated left ventricular cardiomyopathy with LVEF 49% in 2001. Four years later, a deterioration in functional capacity was observed (oxygen consumption peak 11.9 mL/kg per minute), along with a decline in LVEF (33%) and the onset of nonsustained ventricular tachycardia. An implantable cardioverter-defibrillator was implanted in primary prevention. In 2006, the patient presented with marked motor deconditioning and severe muscle atrophy (body mass index, 15.5 kg/m^2^). She experienced progressive increased need for diuretic therapy, leading to advanced HF and required urgent heart transplantation in 2006, without cardiac complications reported. Moreover, a diagnosis of a severe form of EDMD was made and the disease course has been marked by significant disability, with the patient requiring substantial assistance in daily activities.

Patient III.1, now 33 years old, developed mild arrhythmic burden between 2012 and 2017, with short runs of supraventricular tachycardia and nonsustained ventricular tachycardia in the absence of abnormalities at ECG and echo. Bisoprolol was, therefore, initiated, leading to a complete resolution of nonsustained ventricular tachycardia. In 2022, echocardiography documented a mildly reduced LVEF (49%) in the absence of overt HF (New York Heart Association class I). A recent cardiac magnetic resonance was prematurely interrupted due to a cutaneous allergic reaction to gadolinium and was non-contributory. She remains clinically stable and under close follow-up to detect further clinical or instrumental changes that may warrant implantable cardioverter-defibrillator placement. Neurological evaluation confirmed LGMD type 1B, characterized by mild-to-moderate proximal muscle weakness and a waddling gait, associated with mildly elevated creatine kinase (300–600 U/L). Nevertheless, she remains fully ambulatory, professionally active, and independent in all activities of daily living.

This study represents one of the longest clinical follow-ups of a family with a specific *LMNA* variant. The *LMNA c.960del* variant is classified as a frameshift mutation and results in a truncated *LMNA* protein, associated with early onset electrical abnormalities and progressive cardiomyopathy. *LMNA*, in particular for truncating variants, is a well-known arrhythmogenic gene and one of the first in which arrhythmic risk was recognized irrespective of LVEF. In addition to arrhythmias, progression to HF represents a major clinical concern, as reported in previous studies.^3^ Individuals carrying *LMNA* variants may present with either an isolated cardiac phenotype or a combined cardiac and skeletal muscle involvement, most commonly manifesting as EDMD or LGMD: missense variants are more frequently associated with EDMD, whereas truncating variants are more commonly linked to LGMD.^4^ However, in this family, the phenotype was characterized by a broad spectrum of skeletal muscle involvement, ranging from LGMD to EDMD.

An important lesson from this case is that severe extracardiac manifestations should not be considered an absolute contraindication to heart transplantation. Patient III.4, who presented with advanced EDMD, underwent heart transplantation, and this decision proved appropriate after 25 years of follow-up. This observation underscores that advanced HF therapies may be appropriate in selected laminopathy patients, even in the presence of neuromuscular disease.

Moreover, systematic family screening, initiated after the proband’s sudden death, enabled early identification of at-risk relatives, facilitating timely surveillance and interventions. Furthermore, genetic counseling proved essential in guiding clinical decisions, enhancing risk awareness, and supporting informed reproductive choices.

The precise pathophysiologic mechanisms underlying *LMNA*-related cardiomyopathy remain incompletely understood. Proposed pathways include activation of the p38α mitogen-activated protein kinase, which has led to the development of selective inhibitors, but clinical trials have to date been unsuccessful.^5^ Nevertheless, preclinical studies investigating RNA-targeted strategies (antisense oligonucleotides, exon skipping) and genome editing approaches (CRISPR/Cas9 [clustered regularly interspaced short palindromic repeats/clustered regularly interspaced short palindromic repeat–associated protein 9]) hold promise for future targeted therapies.

In conclusion, this 25-year follow-up of the MDDC1 family carrying the *LMNA c.960del* variant highlights the marked intrafamilial variability, ranging from isolated cardiomyopathy, often progressing to HF, to overlapping myopathic syndromes such as LGMD and EDMD. Despite sharing the same pathogenic variant, affected individuals exhibited distinct trajectories, underscoring the clinical complexity of laminopathies and the need for multidisciplinary management, long-term surveillance, and further elucidation of how gene–environment interactions and polygenic architecture contribute to phenotypic variability, even in disorders caused by strong-effect *LMNA* mutations.

## ARTICLE INFORMATION

### Acknowledgments

The authors thank all the patients and their families.

### Disclosures

None.
